# Comparison of Survival in Patients with Isolated Peritoneal Carcinomatosis from Colorectal Cancer Treated with Cytoreduction and Melphalan or Mitomycin-C as Hyperthermic Intraperitoneal Chemotherapy Agent

**DOI:** 10.1155/2018/1920276

**Published:** 2018-12-13

**Authors:** Arkadii Sipok, Armando Sardi, Carol Nieroda, Mary Caitlin King, Michelle Sittig, Vadim Gushchin

**Affiliations:** Surgical Oncology Department, Mercy Medical Center, Baltimore, Maryland, 21202, USA

## Abstract

**Background:**

The role of hyperthermic intraperitoneal chemotherapy (HIPEC) for peritoneal carcinomatosis (PC) from colorectal cancer (CRC) is debated. Melphalan as a perfusion agent has also demonstrated survival benefit in other recurrent and chemoresistant malignancies. Thus, we hypothesize that melphalan as a HIPEC agent may improve overall survival (OS) and progression-free survival (PFS) in patients with PC from CRC.

**Methods:**

A retrospective review of a prospective database of 48 patients who underwent optimal CRS (CC-0/1) and HIPEC from 2001-2016 was performed. Nineteen had CRS/HIPEC with melphalan (group I) and 29 with mitomycin-C (group II). Survival was estimated using the Kaplan-Meier method. Cox regression was used for multivariate analysis. Perioperative variables were compared.

**Results:**

Mean age at CRS/HIPEC was 53±10 years. Median peritoneal cancer index (PCI) was 17 vs 13 in groups I and II, respectively (p=0.86). PCI≥20 occurred in 9 (47%) and 13 (45%) patients in groups I and II, respectively. Positive lymph nodes were identified in 8/19 (42%) vs 12/29 (41%) in groups I and II, respectively (p=0.73). Multivariate analysis identified PCI≥20 as a predictive factor of survival (HR: 7.5). Median OS in groups I and II was 36 and 28 months, respectively (p=0.54). Median PFS in groups I and II was 10 and 20 months, respectively (p=0.05).

**Conclusions:**

CRS/HIPEC with MMC had longer median PFS in PC from CRC. PCI≥20 was the only independent predictive factor for survival. Until longer follow-up is available, we recommend using MMC in CRS/HIPEC for PC from CRC. Further prospective randomized studies are necessary.

## 1. Introduction

Cytoreductive surgery (CRS) combined with hyperthermic intraperitoneal chemotherapy (HIPEC) has well established predictors, described techniques, and indications for the treatment of peritoneal surface malignancies; however, the HIPEC portion of the treatment is not standardized by methods or chemotherapeutic agents. This is especially true in the treatment of peritoneal carcinomatosis (PC) from colorectal cancer (CRC).

Mitomycin-C (MMC) is currently the most common and standard perfusion agent in the United States. In 2013, the American Society of Peritoneal Surface Malignancies (ASPSM) published standardized guidelines for the use of HIPEC in CRC in the United States [1]. These guidelines require (1) a closed method of HIPEC using (2) mitomycin-C (MMC) at a (3) total dose of 40mg (4) initially delivered at 30mg for 60 minutes and adding 10mg for the last 30 minutes, (5) diluted in 3L of perfusion solution, and (6) heated to 42°C (7) for a total perfusion time of 90 minutes [1]. Although a variety of other chemotherapeutic agents have been used, including cisplatin, carboplatin, oxaliplatin, doxorubicin, 5-fluorouracil, and irinotecan [2, 3], there is no clear best choice among them. Therefore, the search is ongoing.

Melphalan may be a good HIPEC perfusion agent due to its several advantages, including better potentiation of the drug by hyperthermia with lower absorption from the abdominal and pelvic regions to blood during the procedure, resulting in lower toxicity potential [4, 5]. The clinical efficacy of melphalan as a second-line HIPEC agent has been demonstrated by our center in patients with various primaries who failed previous CRS/HIPEC treatment with MMC, MMC/doxorubicin, MMC/cisplatin, and doxorubicin/cisplatin [6].

We hypothesize that if melphalan is an effective HIPEC agent option for recurrent PC after previous CRS/HIPEC, it may offer survival benefit if used at the time of initial CRS/HIPEC. We compared overall survival (OS) and progression-free survival (PFS) in patients with PC from CRC undergoing initial CRS/HIPEC using either MMC or melphalan as the HIPEC agent.

## 2. Materials and Methods

A retrospective review of a prospective database of 65 patients who underwent CRS/HIPEC for CRC with PC in a single medical center from February 2001 to January 2016 was performed. Inclusion criteria were (1) use of melphalan or mitomycin-C as a HIPEC agent (2) optimal CRS (CC-0/1) and (3) absence of distant metastases at the time of HIPEC. Forty-eight patients were identified for analysis: 19 patients treated with melphalan (group I) and 29 patients treated with MMC (group II). Radiographic imaging and/or biopsies along with clinical presentation supported the preoperative PC diagnosis. From 2001-2012, mitomycin-C was the primary perfusion agent used for PC from CRC at our center. In 2012, one surgeon empirically began perfusing with melphalan because of the drug's success in recurrent and resistant malignancies and dissatisfaction with MMC results in CRC. The other surgeon continued to primarily use mitomycin-c until 2014 when melphalan became the perfusion agent of choice for PC from CRC, at our institution. Both surgeons consistently used the same selection criteria for CRS/HIPEC throughout the study period.

### 2.1. Patient Selection

Imaging studies, such as computed tomography (CT), magnetic resonance imaging (MRI), or fluorodeoxyglucose positron emission tomography (FDG-PET), and diagnostic laparoscopy (when necessary) were performed to estimate the feasibility of complete cytoreduction (CC-0/1). Extensive involvement of small bowel, its mesentery, or the porta hepaticus were contraindications for the procedure. In case of extensive, but potentially resectable disease, or a recent extensive incomplete debulking, patients underwent neoadjuvant chemotherapy and were then reevaluated for the feasibility of achieving a complete cytoreduction.

### 2.2. Surgical Technique and Patient Care

A xiphopubic incision was made under general anesthesia. The peritoneal cavity was inspected and the peritoneal carcinomatosis index (PCI) score was recorded as previously described by Jacquet et al [7]. Patients with presumed PCI ≥20 underwent diagnostic laparoscopy to estimate the feasibility of achievement of complete cytoreduction (CC). The extent of CRS performed was determined by the specific involvement of organs and structures at the time of surgery with the goal to achieve complete (CC-0) or optimal (CC-1) cytoreductions. Only those involved with disease were removed. Common resections included: previous scar and port sites of the anterior abdominal wall, extensive peritonectomies, including parietal, diaphragmatic, abdominal wall, pelvic, as well as visceral, and peritoneal stripping of the omental bursa and porta hepatis. If tumor lesions were impossible to remove from the surface of solid organs, the organ was resected. Commonly resected organs include bowel and liver segments, uterus, ovaries, and gallbladder. After CRS, completeness of cytoreduction (CC) score was estimated and defined as CC-0 if no visible tumor remained within the abdomen or CC-1 if there were residual tumor nodules <2.5mm. Patients with ≥2.5mm of residual tumor (CC-2/3) were excluded from analysis.

Then, HIPEC perfusion was performed using a closed technique with either melphalan (50mg/m^2^) or MMC (40mg) at 42-43°C for 90 minutes. The target temperature of the intraperitoneal chemotherapy solution was 41-42°C, which was achieved by inflow temperatures of 41-43°C. After completion of HIPEC, anastomoses were created and the incision was closed [8].

Following CRS/HIPEC, patients were observed in the intensive care unit for the first 24 hours or until stable and then were transferred to the surgical oncology unit for further observation. Postoperative surgical complications were analyzed using the Clavien-Dindo classification [9].

Clinical follow-up occurred at 3 weeks, 3 months, every 6 months after CRS/HIPEC for 5 years, and yearly after 5 years. OS was defined as the time from CRS/HIPEC to the date of the last contact (censored) or date of death (event). PFS was defined as the time from CRS/HIPEC and the date of the last contact (censored) or date of recurrence (event). Disease recurrence was identified by imaging studies (CT-scan, PET-scan or MRI), elevated biochemical markers (CEA, CA 125, or CA 19-9), and/or clinical presentation (i.e. bowel obstruction). Patients with more than one CRS/HIPEC were included only in PFS analysis from the time of initial CRS/HIPEC to first recurrence.

IBM SPSS 23.0 software package was used (IBM Corp., Armonk, NY USA) for statistical analysis of clinical data. OS and PFS were analyzed using the Kaplan-Meier method. The Log-rank test was used to determine differences between groups and for univariate analysis. Multivariate analysis using Cox regression was performed to exclude confounding variables. Categorical variables were analyzed with the Chi-square test, while continuous variables were analyzed with the Mann-Whitney or t-test. Differences were considered statistically significant if p≤0.05.

## 3. Results

### 3.1. Patient Characteristics

Nineteen patients were treated with melphalan (group I) and 29 patients were treated with MMC (group II). There were 14 (74%) female patients in group I and 13 (54%) in group II (p=0.049). Mean age at the time of CRS/HIPEC was 53±10 years: 52±11 and 51±9 years in groups I and II, respectively (p=0.73). Forty-two (88%) patients had prior surgery for curative intent: 17 (90%) in group I and 25 (86%) in group II. All of these patients received chemotherapy after prior surgery in groups I and II, respectively (p=0.9).  Table 1 shows preoperative patient characteristics.

The primary tumor site was the right colon in 6 (32%) and 8 (28%) patients in groups I and II, respectively (p=0.88). Well, moderately, and poorly differentiated tumors were observed in 2 (11%), 16 (84%), and 1 (5%) patients vs 3 (10%), 17 (59%), and 8 (28%) in groups I and II, respectively (p=0.98).

Synchronous PC was diagnosed in 8 (42%) and 11 (38%) patients in group I and group II, respectively (p=0.43). Six patients underwent CRS/HIPEC as initial treatment [group 1: 2 (11%); group 2: 4 (14%)]. Of these, 3 received neoadjuvant chemotherapy [group I: 1 (5%); group II: 2 (7%)] (p=1). A total of 52 CRS/HIPEC procedures were performed in all patients: 1 patient in group I underwent 2 procedures and 2 patients in group II underwent 2 and 3 procedures, respectively.

### 3.2. Treatment and Outcomes

Median length of CRS/HIPEC was 9 hours (range: 5-14) in both groups. The median preoperative PCI was 17 (range: 3-35) and 13 (range: 3-39) in groups I and II, respectively (p=0.86). All patients had optimal cytoreductions (CC-0/1). Fourteen (74%) patients in group I and 25 (86%) patients in group II had CC-0 cytoreductions (p=0.28).

Sixteen (84%) patients in group I and 22 (76%) patients in group II had lymph node (LN) metastases (p=0.72). Median number of LN resected during CRS/HIPEC was 26 (range: 6-119) and 17 (range: 1-53) in groups I and II, respectively (p=0.043). LN metastases identified at CRS/HIPEC were identified in 8 (42%) patients in group I and 12 (46%) patients in group II (p=0.73).

Median hospital stay was 11 days (range: 9-17) in group I and 10 days (range: 5-59) in group II (p=0.89). Grade II/III/IV postoperative complications occurred in 7/6/0 patients in group I and in 4/1/3 patients in group II, respectively (p<0.01). Grade IV postoperative complications occurred in 3 (10%) patients in group II. One patient had a myocardial infarction, gastro-jejunostomy anastomic failure requiring reoperation, and a left upper quadrant abscess. The second patient had a small bowel fistula with peritonitis complicated by sepsis and respiratory failure, which required complex treatment including antibacterial therapy, intubation, and reoperation. The third patient underwent reoperation for a left gastric artery hemorrhage. Bone marrow toxicity was observed in 10 (53%) patients in group I and 3 (10%) in group II (p<0.01). There was no 30-day postoperative mortality.

### 3.3. Survival

**Figure 1:**
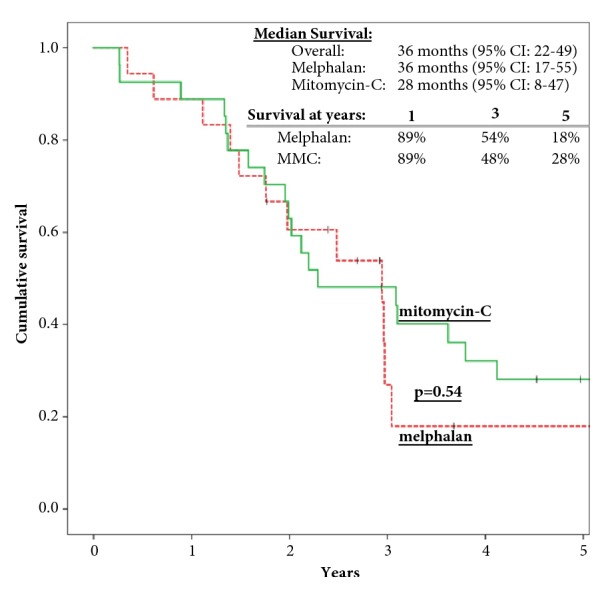
Kaplan-Meier curves demonstrate overall survival of patients with PC from CRC treated with CRS/HIPEC. OS was calculated in 45 patients (patients with >1 CRS/HIPEC were excluded).

At the time of the analysis, 7/19 (37%) patients in group I were alive: 5 alive with disease and 2 with no evidence of disease. In group II, 6/29 (21%) patients were alive, all without evidence of disease. The mean follow-up was 38±13 months in group I and 57±11 months in group II (p=0.02). Median OS was 36 (95% CI: 17-55) and 28 months (95% CI: 8-47) in groups I and II, respectively. OS at 1, 3 and 5 years is 89%, 54%, and 18% in group I and 89%, 48%, and 28% in group II, respectively (p=0.54) (Figure 1).

**Figure 2:**
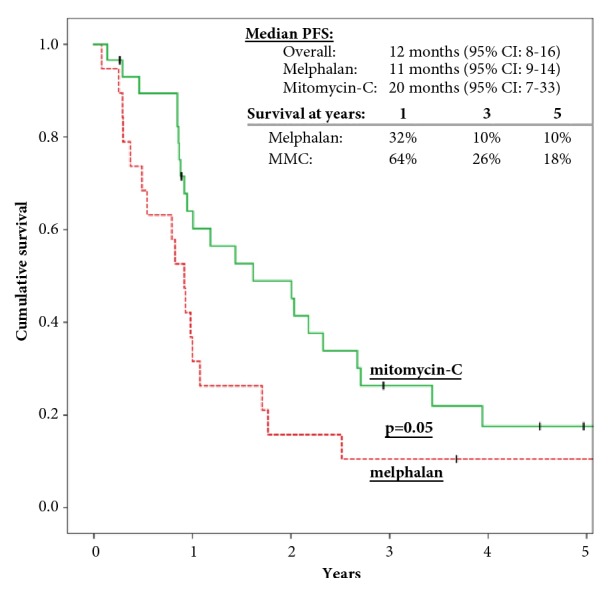
Progression-free survival of patients with PC from CRC treated with CRS/HIPEC.

Seventeen (89%) patients in group I and 22 (75%) patients in group II had disease recurrence. Median PFS was 10 (95% CI: 6-13) and 20 months (95% CI: 9-39) in groups I and II, respectively. PFS at 1, 3 and 5 years is 32%, 10%, and 10% in group I and 64%, 26%, and 18% in group II, respectively (p=0.05) (Figure 2).

In the univariate analysis, CC-0 (vs CC-1) and preoperative PCI<20 were identified as variables associated with better PFS and OS; however, in the multivariate analysis only preoperative PCI <20/≥20 was an independent factor for progression and survival (Table 2) (Table 3).

## 4. Discussion

Melphalan was never compared to MMC as HIPEC agent in regards of survival outcomes in patients with PC from CRC. HIPEC agents for PC from CRC vary with the institution and/or surgical group performing the procedure, making the analysis of outcomes difficult. In the United States, the most common drug for these patients is MMC. However, the use of MMC has also varied in doses (15-60mg/m^2^), temperature (40-43°C), and duration of exposure (30-120 minutes) depending on the institution [1]. Recently, the American Society of Peritoneal Surface Malignancies (ASPSM) released guidelines for HIPEC in patients with PC from CRC which established MMC (40mg at 42°C for 90 minutes) as the standard intraperitoneal perfusion agent and drug of choice for PC from CRC [1]. We followed these recommendations when MMC was used.

It is important to note that the American Society of Peritoneal Surface Malignancies did not publish consensus guidelines for HIPEC in PC from CRC until 2014 and there is very limited data testing these guidelines, which recommend MMC for 90 minutes. Initially, we consistently used MMC as the primary HIPEC perfusion agent for PC from CRC. Empirically, we noticed that MMC outcomes were not as good as we expected, with seemingly quick time to progression. Therefore, based on the pharmacologic and clinical properties, as well as its success in aggressive and resistant malignancies, one surgeon began using melphalan in 2012, while the other HIPEC surgeon at our center continued to use mitomycin-C. At the 2013 regional meeting of the Mid-Atlantic Chapter of the American Society of Peritoneal Surface Malignancies, the use of melphalan as an alternative to MMC was discussed and agreed upon by the physicians as a valid method to try and improve outcomes in PC from CRC. Thus, in 2014, melphalan became the primary perfusion agent for PC from CRC at our center for both surgeons. Melphalan was chosen based on its proven success in recurrent, resistant, and aggressive tumors, such as melanoma and sarcoma with only one time applications [16, 17]. Our group has also demonstrated melphalan to be effective in subsequent HIPEC procedures in patients who recurred after previous CRS/HIPEC procedures with MMC, MMC/doxorubicin, MMC/cisplatin, or doxorubicin/cisplatin, we demonstrated [6]. Pharmacologically, as an alkylating agent, along with ifosfamide and cyclophosphamide, it has the highest potentiation by heat [5]. Melphalan was the most appropriate choice since ifosfamide and cyclophosphamide are prodrugs which require activation by liver microsomal enzymes and, thus, might be less cytotoxic to tumor cells if used in intraperitoneal solution. Moreover, when used for intraperitoneal perfusion, melphalan demonstrates a favorable peritoneal fluid to plasma AUC ratio of 35±13 [4]. Therefore, it achieves higher local concentrations of chemotherapy and lower systemic concentrations [6]. Hence, we used melphalan due to its theoretical and empirical benefit for survival.

There are also several pitfalls to using melphalan as a HIPEC agent related to its pharmacokinetic and pharmacodynamic properties. A serious disadvantage is its rapid spontaneous degradation by hydrolysis. Thus, a minimum delivery time of only 20-30 minutes from the pharmacy to the operating room and perfusion into the peritoneal cavity is imperative. Maintaining the proper temperature (<42°C) is critical for the same reason [18]. According to studies by Urano et al., melphalan achieves its highest efficacy at 41.5°C [19]. In our study, melphalan was perfused intra-abdominally with median in-port temperature control of 43.3°C (range: 42.4-46.6). Although melphalan was sometimes perfused at a higher median temperature than recommended which may have effected the efficacy of the drug, it appears that our patients still received an therapeutic dose since 10/19 (53%) experienced complications related to bone marrow toxicity. Therefore, to achieve better results using melphalan as a HIPEC agent, proper planning to minimize delivery time and tight temperature control is necessary. Selecting a proper dosage of melphalan is also important to limit toxicity. Bijelic et al. showed that in patients who underwent CRS/HIPEC with melphalan, grade IV complications were associated with a higher dose of the drug (70mg/m^2^). The authors recommended the use of melphalan at a dose of 60 mg/m^2^ for 60 minutes [4]. We used a dose of 50 mg/m^2^ perfused for 90 minutes, which may explain the absence of grade IV complications in our study. Thus, using melphalan at a lower dose may be safe and reduce the number of postoperative complications attributed to drug toxicity, however; further studies on the dosage and duration of the perfusion agent(s) are necessary to determine the most effective combination to treat PC from CRC.

Few studies are available reporting the surgical and hematological complications associated with melphalan as a HIPEC agent. In our study, melphalan demonstrated a higher hematologic toxicity and lower grade III/IV surgical complications compared to MMC. We observed grade III surgical complications in 6 (32%) patients and no (0%) grade IV complications. Hematologic complications occurred in 10 (53%) patients perfused with melphalan of which 5 (26%) developed neutropenia. All cases of neutropenia were successfully managed with filgrastim. These findings validate our previous study in which grade III/IV surgical complications occurred in 7 of 31 cases (23%), while neutropenia was observed in 9 of 31 cases (29%) after CRS/HIPEC with melphalan in patients with PC from various primaries [6]. In a recent study, Hakeam et al compared hematologic complications after CRS/HIPEC with either melphalan (60 mg/m2) or MMC+cisplatin (30 mg/m2 and 100 mg/m2, respectively) for 60 minutes. Leukopenia was observed in 26% and 17% of patients perfused with melphalan and MMC, respectively (p=0.36). Leukopenia occurred earlier in patients perfused with melphalan vs MMC+cisplatin (p=0.033), however; neutropenia did not occur in study patients, which was suggested by the authors to be a result of using an open technique [20]. More studies are needed to evaluate variables involved in the drug effectiveness and toxicity in patients with PC from CRC.

The role of HIPEC in PC from CRC is debated and only one prospective randomized phase III study comparing CRS/HIPEC with oxaliplatin for 30 minutes + systemic chemotherapy (fluorouracil + leucovorin) vs CRS + systemic chemotherapy (fluorouracil + leucovorin) alone was conducted in France (NCT00769405). The first results of this trial presented at the 2018 American Society of Clinical Oncology meeting showed no statistically significant difference between the groups with median survival of 41.2 months vs 41.7 months in patients treated with CRS/HIPEC + adjuvant systemic chemotherapy versus CRS + adjuvant systemic chemotherapy, respectively (p=1). However, there are many different ways to adjust the HIPEC procedure to achieve superior outcomes, i.e. drug, dosage, time of perfusion, temperature, and surgical technique. Thus, the search of the optimal combination HIPEC technique, including drug choice, continues.

Several studies have reported outcomes for CRS/HIPEC with MMC for PC from CRC with median OS ranging from 13-30 months and up to 48 months with optimal cytoreduction (Table 4). In the 2014 ASPSM study, CRS/HIPEC with MMC demonstrated a significantly positive impact on survival in the multivariate analysis of all patients (HR: 1.40 [95% CI: 1.01-1.94]) over oxaliplatin, but failed to show significant difference in patients with CC-0/1 cytoreductions (HR: 1.24 [95% CI: 0.87-1.76]) [15]. This may suggest MMC is more cytotoxic to CRC tumor cells. Similarly, in our study, no statistically significant difference was observed in OS between MMC and melphalan. However, MMC had a longer PFS than melphalan (median: 20 vs 11 months, p=0.05). We did not observe a difference in survival by perfusion drug in either uni- or multivariate analysis. Other studies have identified PCI, CC score, LN metastases, and adjuvant chemotherapy as predictive factors for survival [12, 21]; however, our multivariate analysis only found PCI (<20 or ≥20) to be a predictive factor. Although some studies identified PCI as a prognostic factor for long term survival and it is often used as a criterion for patient selection, there is no firm consensus on patient selection for CRS/HIPEC in PC from CRC based on PCI score [22]. The key prognostic factor is the completeness of cytoreduction [12, 22], lending to the importance of referrals to high-volume, specialized centers [10, 11]. Patients in our study were selected for CRS/HIPEC if a complete cytoreduction was deemed feasible, based on imaging studies and confirmed by exploratory laparoscopy when needed. The effect of HIPEC is on the residual disease after cytoreduction. Although higher PCI scores (>20) can make achieving a CC score of 0 or 1 more challenging, a complete cytoreduction was achieved in 100% of the patients in our study, including those with PCI>20. In addition, the number of patients with PCI>20 in each group was statistically equal (45% MMC vs 47% melphalan, p=1) and the majority of patients had PCI<20 (median PCI: 17 MMC vs 13 melphalan, p=0.86). Preoperative tumor burden plays an important role in patient survival outcomes, however; if a complete cytoreduction is feasible, PCI should not be considered a contraindication to proceed with CRS/HIPEC.

There are several possible explanations why we did not find any difference in survival outcomes between melphalan and MMC groups in this study. It is a retrospective study with relatively small number of patients and low statistical power to detect clinically relevant difference. In addition, the melphalan was perfused at a higher median perfusion temperature than indicated for this drug. Also, the melphalan group had a significantly shorter mean follow-up (38±13 months) compared to the MMC group (57±11 months). Therefore, further studies with better temperature control and a larger sample size with longer follow-up are needed.

The interest of using melphalan as a perfusion agent in patients with PC from CRC is strong. A phase II randomized trial, entitled “Comparing Cytoreductive Surgery and Hyperthermic Intraperitoneal Chemotherapy (CRS/HIPEC) Using Mitomycin-C versus Melphalan for Colorectal Peritoneal Carcinomatosis” (NCT03073694), was recently registered in 2017 by the University of Kansas Medical Center. An objective of the trial is to compare the toxicity profiles of these drugs. No results have been published yet.

## 5. Conclusions

Although OS was not statistically different between patients with PC from CRC treated with CRS/HIPEC with either melphalan or MMC, PFS was statistically longer in patients perfused with MMC. PCI was the only independent predictive factor for survival in multivariate analysis. Therefore, we have discontinued melphalan as a primary HIPEC agent for PC from CRC. Until further studies are available, we recommend using mitomycin-C. Further prospective studies on the role of melphalan are needed.

## Figures and Tables

**Table 1:** Patient characteristics.

	Melphalan (n=19)	Mitomycin-C (n=29)	p-value
**Gender **			**0.048**
Male, n (%)	5 (26)	16 (55)	
Female, n (%)	14 (74)	13 (45)	
Number of previous surgeries with curative intent			0.99
0 (%)	2 (10)	4 (14)	
1 (%)	15 (80)	21 (72)	
>1 (%)	2 (10)	4 (14)	
Median LN resected from previous surgery, (range)	15 (0-43)	14 (0-145)	0.87
LN metastases at the time of previous surgery, n (%)	12 (71)	18 (67)	1
Adjuvant chemotherapy after previous surgery, n (%)	17 (90)	25 (86)	0.9
Location of tumor origin			0.88
Right, n (%)	6 (32)	8 (28)	
Left, n (%)	13 (68)	21 (72)	
Peritoneal carcinomatosis occurrence			0.63
Synchronous, n (%)	8 (42)	11 (38)	
Metachronous, n (%)	13 (58)	18 (62)	

**At the time of CRS/HIPEC**			

NACT (before HIPEC), n (%)	1 (5)	2 (7)	1
Mean age at the time of CRS/HIPEC, years (SD)	52±11	51±9	0.73
Median time from diagnosis to CRS/HIPEC, months (range)	22 (0-82)	16 (0-97)	0.89
Median preoperative PCI (range)	17 (3-35)	13 (3-39)	0.86
PCI≥20, (%)	9 (47)	13 (45)	1
Median LOS, minutes	569	580	0.78
Median LN resected from CRS/HIPEC, n (range)	26 (6-119)	17 (1-53)	0.07
Lymph nodes metastases, n (%)	8 (42)	12 (46)^†^	0.73
Median length of stay in hospital, days (range)	11 (7-17)	10 (5-59)	0.89
Histopathology			0.98
Well differentiated, n (%)	2 (11)	3 (10)	
Moderately differentiated, n (%)	16 (84)	17 (59)	
Poorly differentiated, n (%)	1(5)	8 (28)	
Not reported, n (%)	0 (0)	1 (3)	

NACT: neoadjuvant chemotherapy; LOS: length of surgery; ^†^calculated in 26 patients (not reported in 3 patients).

**Table 2:** Univariate and multivariate analysis of survival in patients with colorectal cancer, treated with melphalan or mitomycin-C as a HIPEC agent.

		Univariate		Multivariate	
Characteristics	n	Median survival months (95%CI)	p-value	HR (95%CI)	p-value
Agent			0.54		0.24
Melphalan	18	36 (17-55)		1.66 (0.7-3.95)	
Mitomycin-C*∗*	27	28 (8-47)			
Age			0.42		0.24
≤53*∗*	31	37 (23-51)			
>53	14	30 (11-49)		1.62 (0.72-3.64)	
CC score			0.026		0.69
CC-0*∗*	37	36 (24-49)			
CC-1	8	24 (3-47)		1.25 (0.43-3.64)	
**PCI**			**<0.001**		**<0.001**
≤20*∗*	24	46 (29-64)			
>20	20	18 (12-24)		7.5 (2.76-20.4)	
LN status			0.49		1
no LN metastases*∗*	22	37 (35-39)			
LN metastases	19	27 (18-35)		1 (0.44-2.27)	

HR: hazard ratio; CI: confidence interval; PCI: peritoneal carcinomatosis index; LN: lymph nodes; *∗*used as reference; patients with >1 CRS/HIPEC were excluded from the analysis.

**Table 3:** Univariate and multivariate analysis of progression free survival in patients with colorectal cancer, treated with melphalan or mitomycin-C as a HIPEC agent.

		Univariate		Multivariate	
Characteristics	N	Medial PFS months (95% CI)	p-value	HR (95% CI)	p-value
Agent			0.05		0.17
Melphalan	18	10 (6-13)		1.62 (0.81-3.27)	
Mitomycin-C*∗*	28	24 (9-39)			
Age			0.36		0.89
≤53 years*∗*	32	13 (3-23)			
>53years	14	11 (9-11)		0.95 (0.47-1.96)	
CC score			0.11		0.67
CC-0*∗*	38	13 (3-23)			
CC-1	8	12 (10-16)		1.22 (0.49-3.08)	
**PCI**			**<0.001**		**<0.001**
≤20*∗*	25	28 (18-38)			
>20	20	10 (4-16)		4.52 (2.05-9.95)	
LN status			0.64		0.97
LN metastases	19	10 (9-12)		1.01 (0.5-2.07)	
no LN metastases*∗*	23	12 (9-15)			

HR: hazard ratio; PCI: peritoneal carcinomatosis index; LN: lymph nodes; PFS: progression-free survival; *∗*was used as reference in multivariate analysis; Median age of all patients was 53 years.

**Table 4:** Outcomes in patients with PC from CRC treated with CRS and HIPEC or Early Postoperative Intraperitoneal Chemotherapy.

Study	Year	No of patients	Perfusion agent, dose, length, t°	Median OS (months), (resection status)
Glehen et al. [10]	2004	53 23	MMC: 40-60 mg at 46-48°C for 90 min	13 (all patients) 33 (CC-0)
Glehen et al. [11]	2004	506 271 106	(1) MMC (30/50 mg/m^2^) ± cisplatin (50-100 mg/m^2^) at 41-42.5 °C for 60-120 min (2) oxaliplatin (360-460 mg/m^2^) ± irinoteacan (100-200 mg/m^2^) ± IV 5-FU + leucovorin at 43 °C for 30 min	19 (all patients) 32 (CC-0) 24 (CC-1)
Elias et al. [12]	2010	523 439	(1) MMC (30/50 mg/m^2^) ± cisplatin (50-100 mg/m^2^) at 41°C for 60-120 min (2) oxaliplatin (360-460 mg/m^2^) ± irinoteacan (200 mg/m^2^) ± IV 5-FU + leucovorin at 43 °C for 30 min	30 (all patients) 33 (CC-0)
Verwaal et al. [13]	2008	54	MMC: initial dose of 17.5 mg/m^2^ with additional 8.8 mg/m^2^ every 30 min (maximal dose in total 70 mg/m^2^)at 41-42 °C for 90 min	22 (all patients) 48 (R-1)
Franko et al. [14]	2010	67	MMC: initial dose of 30 mg for 60 min with additional 10 mg after for 40 min	35 (all patients)
ASPSM study [15]	2014	392	MMC: initial dose of 30 mg with additional 10 mg in 60 min at 42°C for 90 minutes	33 (CC-0/1)
This study	2018	19 29	Melphalan MMC	36 (CC-0/1) 28 (CC-0/1)

*MMC*: mitomycin-C; *OS*: overall survival; *R-0*: no gross disease with negative resection margins, *R-1*: no gross disease with positive resection margins; *CC-0*: no visible residual disease; *CC-1*: residual disease <2mm;

## Data Availability

The retrospective data used to support the findings of this study may be released upon application to the Mercy Medical Center Institutional Review Board, who can be contacted at IRB Chair: Ralph Lebron, MD (rlebron@mdmercy.com).
